# Post-Caustic Esophagitis: Updated Clinical Insights and Practical Lessons from Case Illustrations

**DOI:** 10.3390/jcm14238575

**Published:** 2025-12-03

**Authors:** Bogdan Oprita, Alice Elena Diaconu, Bogdan Dinu, Anca Arina Ghiorghiu, Ruxandra Oprita

**Affiliations:** 1 Emergency Department, Clinical Emergency Hospital of Bucharest, 050463 Bucharest, Romania; bogdan.oprita@umfcd.ro (B.O.); alexandru-bogdan.dinu@drd.umfcd.ro (B.D.); 2 Faculty of Medicine, University of Medicine and Pharmacy “Carol Davila”, 050474 Bucharest, Romania; anca-arina-maria.ghiorghiu@rez.umfcd.ro (A.A.G.); ruxandra.oprita@umfcd.ro (R.O.); 3 Gastroenterology Department, Clinical Emergency Hospital of Bucharest, 050463 Bucharest, Romania; 4 Anesthesia and Intensive Care Department, Clinical Emergency Hospital of Bucharest, 050463 Bucharest, Romania

**Keywords:** corrosive ingestion, acid agent, alkali agent, Zargar classification, management of caustic ingestion, esophageal strictures

## Abstract

**Background:** Caustic ingestion represents a severe clinical emergency, leading to variable and often unpredictable outcomes. The severity depends on the nature of the corrosive agent, the amount ingested, and the timing of management. **Summary:** This review provides an updated overview of post-caustic esophagitis, integrating epidemiology, pathophysiology, clinical spectrum, diagnostic strategies, and management principles. Special emphasis is placed on complications such as strictures, fistulas, and long-term nutritional consequences. To illustrate practical aspects, we present two recent cases managed in our department, reflecting the contrasting patterns of acid and alkali injury. A brief reference to our previously published retrospective analysis of 188 patients referred to Clinical Emergency Hospital of Bucharest between January 2003–January 2004 complements this clinical perspective. **Conclusions:** Post-caustic esophagitis remains a major therapeutic challenge. Early endoscopic assessment, individualized management, and multidisciplinary follow-up are crucial to improving patient outcomes. Preventive strategies and public education are equally essential to reduce the incidence of this life-threatening condition.

## 1. Introduction

### 1.1. Definition

Post-caustic esophagitis is simply defined as the totality of esophageal lesions resulting from the ingestion of a caustic substance.

Although the definition is straightforward, the disease exhibits a fluctuating course, determined by superimposed lesions on a background of multiple risk factors—a pathophysiological “roller-coaster” directly proportional to the mechanism of injury, as well as to the type and amount of substance ingested. Therefore, this paper highlights the importance of the prompt, accurate, and comprehensive management of an event that may become fatal, as well as the critical role of multidisciplinary collaboration in influencing the morbidity and mortality associated with post-caustic esophagitis.

In a previous article [1], we reported on the clinical characteristics and overall outcomes of patients with caustic ingestion in our center. That study focused on the epidemiological and general therapeutic aspects. In the present paper, we update the topic and also illustrate it with two newly encountered cases.

### 1.2. Epidemiology

From an epidemiological perspective, the age of onset of the disease displays a bimodal distribution [2,3], with the first and most significant peak occurring between 1 and 5 years of age, when the vast majority of ingestions take place, predominantly accidental in the curious child. The second peak is observed among adolescents and young adults, in whom ingestion may be either accidental or intentional, most often for suicidal purposes. Accidental ingestion generally follows a favorable course, in contrast to suicidal ingestion, which is frequently associated with severe outcomes.

Demographic factors play a significant role in determining the incidence, patterns, and clinical outcomes of caustic substance ingestion.

Accidental ingestions occur more frequently in children, with no consistent sex predominance, whereas intentional ingestions—often associated with self-harm—are reported more frequently among adult females more than males. However, regional variations exist, like higher ingestion rates among men, particularly in contexts involving alcohol co-ingestion [3,4,5].

Race and ethnicity may influence ingestion patterns indirectly through differences in socioeconomic status, accessibility of hazardous household chemicals, and cultural practices regarding storage and handling of caustic agents. While no inherent biological predispositions are identified, certain minority groups demonstrate higher exposure risks due to disparities in housing conditions, education, and access to preventive information.

Psychological factors represent a critical determinant, especially in adolescents and adult populations. Intentional ingestions are strongly associated with underlying psychiatric conditions, including depressive disorders, personality disorders, substance abuse, and acute emotional distress. These cases tend to involve larger volumes or more concentrated chemicals, resulting in notably higher morbidity and mortality.

Socioeconomic status also correlates with ingestion patterns. Low-income households may rely on cheaper, highly concentrated industrial cleaning agents stored in unsecured containers, increasing the risk of accidental exposure. Limited access to health education and delayed medical presentation further worsen outcomes in these populations. Conversely, in high-income environments, accidental ingestion often involves smaller quantities of diluted domestic products, resulting in lower injury severity [3].

Overall, demographic characteristics provide essential context for understanding exposure risks, guiding targeted prevention strategies, and interpreting clinical outcomes in both pediatric and adult populations.

### 1.3. Etiological Agents

As indicated by the definition of the disease, the primary etiological factor is the corrosive substance, which can be broadly categorized into alkaline and acidic agents.

Alkalis are caustic agents with a pH > 7, often odorless, tasteless, colorless, and of viscous consistency. They are commonly found in drain cleaners, toilet cleaners, disinfectants, and household detergents. The most well-known alkali is sodium hypochlorite, also referred to in Romania as “lye”.

Acids are caustic agents with a pH < 7, characterized by a pungent odor and an extremely unpleasant taste. They are generally present in industrial solutions, but can also be found in certain household products, such as disinfectants, dust removers, and toilet bowl cleaners [6,7] (Table 1).

**Figure 1:**
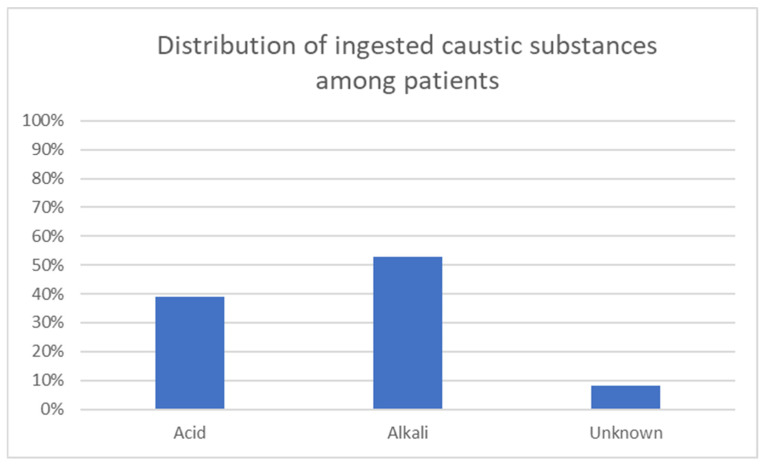
Distribution of ingested caustic substances among patients.

As previously reported in our earlier study, the majority of patients presenting with caustic ingestion were women from rural areas, with approximately 70% of cases being voluntary, typically suicide attempts [1]. The most commonly ingested agents were alkalis, particularly sodium hydroxide, which is widely available in domestic settings in rural regions of our country. Acids were also frequently involved, while a few cases were associated with unidentified substances. The majority of ingestions originated from household products [7] (Figure 1).

## 2. Pathophysiological Mechanism of Injury

### 2.1. Mechanisms of Acid- and Alkali-Induced Injury

The pathophysiological mechanism of injury varies according to the type of corrosive agent ingested, as follows:

Alkali-induced injury: Alkalis react with proteins and lipids upon contact, initiating a saponification process that leads to liquefactive necrosis [5,9], typically lasting 3–4 days and resulting in transmural damage of the affected segment. The process begins with mucosal inflammation, followed by progressive tissue penetration of the substance, which causes thrombosis of small- and medium-sized vessels, ulceration, extensive sloughing, and progressive thinning of the wall. Subsequently, tissue re-epithelialization begins, with the development of granulation tissue and deposition of collagen and fibrin over the course of 1–3 months. The resulting scar formation leads to strictures, directly proportional to the extent of tissue injury.

Acid-induced injury: Acids interact with tissue proteins, converting them into acid proteins, and thereby induce coagulative necrosis through thrombosis of capillaries and small vessels [9,10]. This type of lesion generally prevents deeper tissue penetration of the corrosive substance, thereby limiting transmural involvement in most cases.

Lesions begin within seconds after ingestion and continue to evolve for approximately 4–7 days post-ingestion [11,12], after which the damaged mucosa sloughs off, often complicated by secondary bacterial invasion. Lower esophageal sphincter insufficiency and pyloric spasm, secondary to irritation of neural fibers, contribute on the one hand to reflux of gastric acid into the esophagus, aggravating preexisting lesions, and on the other hand to delayed gastric emptying, which favors stasis of the corrosive agent and additional injury, particularly to the antral mucosa. The process of re-epithelialization typically begins about 3 weeks after ingestion and may continue for several months, depending on the severity of the injury.

Nevertheless, both strong acids and strong bases can cause severe injury, including organ perforation and secondary peritonitis, thereby altering the general pathophysiological course.

### 2.2. Histopathological Classification

- First-degree injury: Superficial lesions confined to the mucosa, which appears erythematous, edematous, with superficial hemorrhagic stigmas. These heal spontaneously without scar tissue formation.
- Second-degree injury: Involvement of both the mucosa and submucosa, characterized by the presence of vacuoles, exudates, and ulcerations. The depth of tissue damage stimulates the process of re-epithelialization and fibroblastic reaction, leading to scar tissue formation.
- Third-degree injury: Transmural damage, extending to perforation, which invariably results in strictures, most often multiple or severe [13].

## 3. Clinical Spectrum

### 3.1. Severity Factors

The extent of injury following caustic ingestion depends on several key factors.

The corrosive properties of the ingested substance play a central role. The most harmful agents are those with a pH below 2 or above 12 [12]. Alkalis, due to their characteristics, are generally ingested in larger amounts compared to acids. They predominantly damage the esophageal mucosa, as they are often viscous liquids and are only partially neutralized by gastric hydrochloric acid. Conversely, acids tend to injure the gastric mucosa for the opposite reasons. Both types of agents may also damage the upper respiratory tract in cases of aspiration.

The concentration and quantity of the ingested agent are directly proportional to injury severity. Ingested volumes greater than 200 mL substantially increase the risk of significant lesions [7], and higher concentrations correspond to more severe damage. Industrial-grade products typically contain higher concentrations of caustic agents, often reaching extreme pH values, while domestic products usually have a pH between 9 and 11, resulting in milder injuries when ingested in quantities below 200 mL.

The physical form of the corrosive agent also influences injury distribution [14]. Solid forms (e.g., tablets, pods, powders) adhere to the mucosa of the oral cavity, oropharynx, and hypopharynx, causing localized lesions. Semi-solid substances pass through the esophagus with difficulty, damaging its mucosa and, depending on the ingested amount, also the gastric mucosa. Liquid caustics traverse the esophagus rapidly and mainly damage the stomach, where the contact time with the mucosa is longer.

Finally, the duration of mucosal contact is a decisive factor. The longer the corrosive agent remains in contact with the mucosa, the more extensive and severe the lesions become, as deeper tissue layers are affected [15]. For example, acids often induce vomiting and aspiration due to their unpleasant taste, limiting contact with the esophageal and gastric mucosa but increasing exposure of the tracheobronchial tree, oropharynx, hypopharynx, and oral cavity, where significant lesions may occur.

Timely presentation to the emergency department following caustic substance ingestion is of critical importance for improving patient outcomes [15,16,17]. Early clinical evaluation enables rapid identification of life-threatening complications, such as airway compromise, perforation, or severe metabolic disturbances, which require immediate intervention. Furthermore, prompt endoscopic assessment [11,12,17] within the optimal diagnostic window allows for accurate grading of mucosal injury and guides appropriate therapeutic decisions. Delayed presentation is associated with a higher risk of missed early complications, progression of tissue necrosis, and suboptimal management, ultimately worsening prognosis. Therefore, educating both the public and healthcare providers about the necessity of early medical evaluation after caustic ingestion represents a key component in reducing morbidity and mortality.

Understanding the pathophysiological mechanisms triggered by caustic substance ingestion is essential for the accurate evaluation and management of affected patients. These mechanisms initiate a cascade of inflammatory responses, vascular compromise, and progressive tissue necrosis, which may evolve rapidly in the first hours after ingestion. Early recognition of these processes is crucial, as the degree of damage correlates directly with the risk of perforation, stricture formation, systemic toxicity, and long-term morbidity.

One of our recent patients ingested approximately 300 mL of an alkaline household cleaning solution. The injury was extensive, involving the oral cavity, pharynx, and upper airways, necessitating emergent intubation and later tracheostomy. Severe circumferential damage was also documented in the esophagus and stomach. This case exemplifies the liquefactive necrosis produced by alkali ingestion, in which deep tissue penetration leads to widespread and transmural injury.

Another patient ingested a smaller volume of a caustic substance, but with an extremely high industrial concentration—namely formic acid. The lesions were localized predominantly to the oral cavity and the esophagus, with severe esophageal injury but relative sparing of the stomach. This pattern reflects the coagulative necrosis typical of acid ingestion, in which tissue damage is more localized, though still potentially severe.

These two cases illustrate how both the type of agent and the concentration and volume ingested influence the pathophysiological mechanism of injury.

Analysis of previously reported cases [1] showed that a significant proportion of patients developed esophageal strictures, particularly those who ingested lye. Gastric strictures were also observed, primarily in the antral region among patients who remained nil per os, and in the mid-gastric region among those who resumed early oral intake. A smaller percentage of patients experienced gastrointestinal perforations.

### 3.2. Clinical Presentation

Despite the risks associated with caustic ingestion, the clinical profile does not always correlate with the severity of the lesions. However, it may be striking at presentation in cases complicated by perforation and major metabolic disturbances, or may become more evident during the dynamic course of the disease.

In most cases, at initial evaluation in the emergency department [8], patients present with psychomotor agitation, complaining of dysphagia and/or odynophagia, retrosternal pain or epigastralgia, hypersalivation, and/or hematemesis. In cases of aspiration, patients may develop dyspnea, tachypnea, and stridor, progressing to severe acute respiratory failure due to edema of the upper airways. Fever, signs and symptoms of shock, peritoneal irritation, or severe and persistent retrosternal pain are highly suggestive of complicated forms with eso-gastric perforation [12].

The most frequent complications include [7,12,17,18] upper gastrointestinal bleeding, manifested by hematemesis and melena, typically 2–4 weeks post-ingestion, indicating erosive and ulcerative lesions of the upper gastrointestinal tract or, in rare cases, arterial fistulas. Additional complications may include fistulization into neighboring structures, such as tracheoesophageal or aortoenteric fistulas, the development of esophageal squamous cell carcinoma long after the event, and—most commonly—esophagogastric strictures. Stricture formation, affecting at least one-third of patients with moderate-to-severe disease, leads to secondary protein–energy malnutrition. Strictures result from re-epithelialization with scar tissue of lesions extending at least to the submucosa. They most commonly occur in the esophagus, with patients presenting to the emergency department approximately two months post-ingestion with dysphagia to solids, or even total dysphagia and/or foreign body retention [11,12,15].

Gastric strictures are less frequent and are more often associated with acid ingestion. Their clinical manifestation may occur months or even years after the initial event, typically presenting as persistent postprandial vomiting due to pyloric stenosis or altered gastric anatomy, caused by pseudodiverticula, scar-induced linitis plastica-like changes, or antral deformity [7].

In addition to the general clinical spectrum described in the literature, and in order to better highlight the clinical course and potential sequelae of caustic ingestion, we present two illustrative cases from our practice. Both patients initially presented in a relatively stable condition from a respiratory and hemodynamic standpoint, although they appeared anxious and frightened, with pharyngeal hyperemia and hypersalivation. The patient who ingested concentrated formic acid was later discharged but subsequently developed severe sequelae, including multiple esophageal strictures and progressively worsening protein–calorie malnutrition, eventually leading to a cachectic state. In contrast, the patient who ingested a large volume of alkaline solution deteriorated rapidly, requiring intensive care admission with endotracheal intubation and subsequent tracheostomy for severe upper airway obstruction and edema. During hospitalization, this patient developed upper gastrointestinal bleeding manifested by hematemesis and melena due to complicated esogastric ulcers, moderate post-hemorrhagic anemia, hospital-acquired pneumonia, pulmonary thromboembolism, and significant fluid and electrolyte disturbances. After discharge, the patient continued to experience severe esophageal strictures and extreme weight loss, raising the suspicion of superior mesenteric artery syndrome. Surgical jejunostomy feeding was therefore considered. More recently, the patient developed an additional complication in the form of a tracheoesophageal fistula.

## 4. Diagnosis

The timing of the initial evaluation plays a decisive role, as both the therapeutic approach and the subsequent prognosis depend on the speed and accuracy of diagnosis. At patient presentation, the priority is the rapid classification of the case into an emergency category; this stage of triage and stabilization is fundamental for guiding and integrating specific investigations.

### 4.1. Clinical Assessment

Anamnesis and heteroanamnesis are essential for evaluating the type of ingested agent, the time of ingestion, the ingested quantity, and any pre-hospital interventions. In the pre-hospital phase [8], particularly in areas with limited medical resources and poor health education, management of caustic ingestion victims is often influenced by myths and empirical practices passed down through popular experience. These include attempts to “neutralize” the caustic agent by inducing vomiting [14], which exposes the digestive mucosa to a second chemical and mechanical aggression, thereby worsening the lesions. Likewise, the administration of dairy products (milk, cream, yogurt), vegetable oils, or other fatty substances is frequently attempted under the false assumption of a protective effect, although such measures only delay access to specialized medical care. Other harmful practices include empirical gastric lavage, ingestion of substances with an opposite pH (acids for alkali ingestion and vice versa), or administration of raw solid foods or activated charcoal in an attempt to “buffer” the corrosive agent. All these unscientifically validated interventions not only fail to provide benefits but may aggravate esophagogastric injury and increase the risk of complications [6,8,14].

The objective clinical examination must always include monitoring of vital parameters; inspection of the oro- and hypopharynx, which may reveal edema, hyperemia, pseudomembranes, erosions, ulcers, or even necrosis; assessment of the skin for “drip lesions” secondary to contact with the corrosive agent during and after ingestion; evaluation of respiratory status through recognition of tachypnea, dyspnea, respiratory distress, laryngospasm, or bronchospasm, which may guide the need for emergent or rescue airway protection; and evaluation of cardiovascular status, with hypotension and tachycardia considered indicators of severe shock. Finally, clinical signs of gastrointestinal perforation (e.g., peritoneal irritation, subcutaneous emphysema) must be sought, as they represent a clear indication for life-saving surgical intervention [8].

### 4.2. Paraclinical Evaluation

#### 4.2.1. Laboratory Tests

In the emergency setting, the biological assessment must include a complete panel of tests such as complete blood count, serum electrolytes, serum biochemistry (including liver function tests and renal function tests), C-reactive protein, blood gas analysis (ASTRUP), and toxicology screening. Coagulation profile testing may also be useful when hospitalization is required and central venous catheter placement is being considered for vascular access in severe cases. During hospitalization, patients should undergo dynamic biological monitoring, with the basic analyses supplemented as necessary by bacteriological tests, procalcitonin, portages, blood group and Rh typing, CK, CK-MB, and D-dimer levels depending on the associated complications [5,8,15].

Initially, laboratory tests may be normal [8,12], or they may reveal a minimal inflammatory syndrome manifested by leukocytosis with or without neutrophilia and elevated C-reactive protein, secondary to caustic burns. In moderate-to-severe forms, disturbances of acid–base balance and hydro-electrolytic status may appear, together with a marked inflammatory response and post-hemorrhagic anemia in patients presenting with hematemesis. In cases of transmural injury, laboratory signs of shock are typically present, such as pronounced neutrophilic leukocytosis, markedly elevated C-reactive protein, severe metabolic lactic acidosis, azotemia, hepatocellular injury, and thrombocytopenia.

Throughout hospitalization, laboratory parameters evolve depending on disease progression and the onset of complications.

#### 4.2.2. Imaging

The most important radiological method is computed tomography (CT) [19,20,21,22], which is superior to both plain radiography and MRI. Emergency contrast-enhanced CT of the chest and abdomen is performed to assess the extent of necrosis, using the following lesion classification system:

- Grade 1: No significant CT changes.
- Grade 2: Esophageal and/or gastric wall edema with contrast enhancement, associated with thickening of the adjacent fat.
- Grade 3: Lack of post-contrast enhancement, suggestive of transmural necrosis; presence of perforations, mediastinitis, pneumoperitoneum, etc.

Based on CT findings, patients are stratified into those requiring surgical intervention and those suitable for non-surgical management, with or without hospitalization, depending on the case.

#### 4.2.3. Endoscopy

Upper digestive endoscopy is the gold standard for diagnosis, as it determines the need for hospitalization, guides subsequent therapeutic planning, and allows estimation of morbidity and mortality from the time of presentation. However, upper digestive endoscopy can only be safely performed within the first 48 h post-ingestion—ideally within the first 24 h—since after this period the risk of perforation increases significantly due to tissue friability, with a peak risk between days 5 and 15 post-ingestion [12].

In patients who ingested a very small amount of a weakly corrosive substance, present only minimal lesions in the oral cavity, are clinically stable, and remain asymptomatic, endoscopy may be omitted [11,17].

Absolute contraindications for upper digestive endoscopy include lack of patient consent, evidence of perforation, hemodynamic instability, and severe respiratory insufficiency.

Endoscopic severity grading is based on the Zargar classification [13] as follows (Table 2).

Thus, as we can observe, grades 0–IIA are associated with mild, superficial lesions with a favorable medium- and long-term prognosis, and the lowest mortality rate. Grades IIB and IIIB present more extensive lesions, both in surface area and depth, and may be complicated in the medium term by upper gastrointestinal bleeding, fistula formation, systemic complications such as metabolic acidosis, infections, malnutrition, and even shock (hypovolemic, septic, or hemorrhagic). In the long term, these patients remain at risk of developing esophageal carcinoma, and all survivors eventually develop strictures. Grade IV corresponds to cases complicated by perforation, with surgical intervention being mandatory; the prognosis is poor and the survival rate low.

Endoscopic evaluation is generally diagnostic, but it may also be therapeutic in cases of gastrointestinal bleeding that lack self-limiting characteristics.

Endoscopic ultrasound (EUS)—can be helpful in assessing the depth of lesions but is not routinely used.

#### 4.2.4. Other

Radiological examination—may be useful when computed tomography cannot be performed, as it can demonstrate the presence of pneumomediastinum or pneumoperitoneum.

Magnetic resonance imaging (MRI)—Compared to CT, it does not provide additional information, has higher costs, and is less accessible in some medical centers. Nevertheless, it may be used in selected patients when further data are required, particularly in those with contraindications to iodine-based contrast agents [15].

As previously reported in our earlier study [1], among 188 patients presenting to the emergency department, 159 underwent endoscopic evaluation after caustic ingestion. Of these, 50 patients exhibited grade I lesions, 82 had grade II lesions, and 27 presented with grade III lesions. In the two newly reported patients, laboratory tests at admission were within normal limits and fluctuated only in relation to late complications, including inflammatory syndrome, anemia, and electrolyte disturbances. Endoscopic assessment classified the patient who ingested concentrated formic acid as Zargar grade IIB, whereas the patient with ingestion of a large volume of alkali solution was graded as Zargar IIIB. Contrast-enhanced CT of the chest, abdomen, and pelvis was performed repeatedly, demonstrating esophageal and gastric wall thickening without signs of perforation. Additional complications included pulmonary embolism, pneumonia, and pleural effusion. Dynamic ENT fiberoptic examinations were also performed to monitor the upper airways due to edema and necrosis.

## 5. Management

### 5.1. Prevention

Prevention is the most important and necessary measure for avoiding this pathology. It begins with educating the population about the risks associated with accidental ingestion of caustic substances, supervising children, the elderly, and individuals with impaired mental capacity, and limiting their access to hazardous substances. It also includes addressing psychiatric comorbidities that may lead to intentional ingestion for suicidal purposes, as well as storing corrosive agents in their original containers to ensure easy identification and to avoid confusion with food or beverages. Furthermore, public education regarding first aid measures for victims of caustic ingestion is essential, with emphasis on avoiding empirical interventions that may compromise prognosis and disease evolution.

### 5.2. Therapeutic Management

Therapeutic management begins in the emergency department with patient stabilization, assessment of lesion severity, identification of complications, and triage toward medical, intensive care, or surgical services, as appropriate.

#### 5.2.1. Supportive Measures Instituted upon Emergency Department Admission

General measures include external decontamination, neutralization and dilution, complete clinical and laboratory evaluation, respiratory support, fluid resuscitation, and pain management [15].

External decontamination and neutralization

These apply to all patients in order to limit the duration of contact between caustic substances and tissues and to prevent accidental exposure of medical staff to the corrosive agent during patient handling. Medical personnel must wear protective equipment (gown, mask, gloves, and face shield, if necessary), remove contaminated clothing, and wash the skin with water and soap to eliminate biological secretions (e.g., saliva, vomit, blood) and residual corrosive substances.

External decontamination represents a critical component of the initial management protocol for patients presenting after caustic substance ingestion. Although the primary injury is internal, external contamination of the skin, ocular and perioral region, or clothing may occur during ingestion or attempted expulsion, and requires immediate intervention. The selection of decontamination agents and the underlying rationale depend on the type of substance ingested, its chemical properties, and the interval between exposure and medical presentation [8].

For alkaline agents, external decontamination consists primarily of copious irrigation with water or normal saline for 15 to 60 min, as early dilution effectively reduces ongoing tissue damage. Neutralizing these substances with acidic solutions is strictly contraindicated, as exothermic reactions can worsen cutaneous injury. In liquid ammonia exposure, crystallization may occur during irrigation; these crystals must be subsequently removed to prevent cryogenic injury.

For acidic agents (such as hydrochloric, sulfuric, or acetic acid concentrate), the recommended approach is similarly based on immediate and continuous irrigation with water or isotonic solutions but for lesser time such as 15 to 30 min. Attempted neutralization with alkaline compounds is avoided due to the risk of heat production and possible extension of tissue injury. In case of sulfuric acid, decontamination should begin with a slow, low-volume water rinse, with the flow rate increased gradually to avoid a violent exothermic reaction upon abrupt contact with water.

In cases involving oxidizing agents (e.g., bleach, peroxides) or hydrocarbon-containing compounds, irrigation with water remains the safest and most effective method. Specialized decontamination products may be considered only when supported by toxicology guidelines and when they do not pose additional thermal or chemical risks. Irrigation of sodium hypochlorite removes both its alkalinity and oxidative activity, while dilution of concentrated hydrogen peroxide decreases its oxidative burden.

For solid caustic agents (e.g., sodium hydroxide pellets, calcium oxide), careful mechanical removal of solid particles prior to irrigation is essential to prevent activation by water.

Phenols pose a risk of transcutaneous absorbtion; therefore, irrigation with polyehylene glycol is preferred, with prolonged water irrigation as an alternative when PEG is unavailable.

Ocular contamination is common in patients who vomit after caustic ingestion. In such cases, copious and continuous irrigation with water or salide is required for 30–120 min or until the conjunctioval pH returns to normal [6].

The timing of external decontamination is crucial. Procedures initiated within the first minutes after exposure significantly reduce the extent of dermal injury and prevent secondary complications. Even when patients present after a considerable delay, residual contamination (particularly in hair, skin folds, or clothing fibers) may still be present; therefore, irrigation should be performed unless contraindicated. All contaminated clothing should be removed and handled using protective equipment to prevent secondary exposure of healthcare personnel [8].

Overall, external decontamination aimed at dilution—not chemical neutralization—remains the safest and most evidence-based approach, regardless of the substance ingested, particularly when the exact timing of exposure is uncertain.

The administration of neutralizing agents such as substances with opposite pH, activated charcoal, or syrup of ipecac is contraindicated, as these interventions bring no benefit and may induce vomiting, exposing tissues to a second chemical and mechanical aggression [8]. They may also cause thermal injury by increasing local temperature through chemical reactions, thereby raising the risk of perforation, and can hinder proper visualization of mucosal lesions during endoscopy (e.g., activated charcoal may adhere to the mucosal surface, masking lesions). For the same reasons, induction of emesis is contraindicated [12,16].

Placement of nasogastric tubes is contraindicated, as it can cause additional lesions or even perforation. Similarly, gastric lavage via nasogastric tube is contraindicated, as it provides no benefit, given that mucosal lesions occur almost instantly after ingestion.

Respiratory support

Patients with caustic ingestion may present with significant lesions of the oral cavity, pharynx, and upper airways, leading to secondary edema, airway obstruction, respiratory distress, and even respiratory arrest. Therefore, the primary priority at presentation is airway evaluation and securing airway patency. The first step is assessment using nasofibroscopy, although this may not be available in all centers. Depending on the findings, emergent or rescue airway protection should be performed via orotracheal intubation under direct visualization of the vocal cords. In cases of severe upper airway lesions with marked edema precluding safe orotracheal intubation, cricothyrotomy must be performed [8,16].

In mild cases without respiratory distress, oxygen therapy via nasal cannula or face mask is sufficient. These patients usually have a favorable prognosis and may require no more than 48 h of post-ingestion medical observation.

Fluid resuscitation

At least two peripheral venous lines should be secured; in severe cases, central venous access is required. Resuscitation includes administration of crystalloid solutions, blood products in cases of significant hemorrhage, vasopressors for patients in shock, correction of acid–base imbalance, proton pump inhibitors to reduce gastric acidity and stress ulcers, and standard analgesics (with avoidance of nonsteroidal anti-inflammatory drugs, which may promote additional ulcerative lesions of the gastrointestinal tract) [8].

Routine administration of antibiotics or corticosteroids is contraindicated, as these do not reduce the risk of stricture formation and may increase the risk of infection, perforation, and bleeding. Antibiotic therapy is reserved for selected cases complicated by infection or shock [16].

After stabilization, patients should undergo radiological and endoscopic evaluation.

In mild cases, with minimal lesions and good general condition, patients may be discharged [7,12,15] with home treatment including analgesics, gastric antisecretory drugs, hyaluronic acid and sodium alginate preparations, and sucralfate.

#### 5.2.2. Specialized Therapeutic Management

Patients classified as Zargar grade I or IIA should be admitted to a clinical ward and monitored for 24–48 h [7,12,13,15]. They should receive standard analgesics, gastric antisecretory agents [23], and parenteral nutrition, followed by gradual reintroduction of oral feeding. Long-term endoscopic follow-up is required to monitor for potential development of esophageal carcinoma [13].

Moderate-to-severe and severe cases require admission to an intensive care unit, with strict monitoring and treatment, as the risk of developing complications is very high and mortality increases in direct proportion to the severity of lesions. Surgical intervention must be considered both at admission and during the clinical course. The appearance of severity markers such as severe leukocytosis, intense or persistent abdominal pain, signs of peritoneal irritation, need for sustained ventilatory support, shock, acute kidney injury, severe metabolic acidosis, and thrombocytopenia should prompt repeat imaging by computed tomography and surgical reassessment. Endoscopic resources are limited in this phase due to the elevated risk of perforation and, secondarily, death. In surgically indicated cases, exploratory laparotomy is preferred over laparoscopy, as it provides better visualization of the posterior gastric wall and retroperitoneal space [7,12,24].

A cornerstone of management is the assessment of the patient’s nutritional status. A complete digestive rest regimen (nil per os) is recommended for 48 h, after which a liquid diet may be introduced if the patient is able to swallow saliva and does not present with vomiting. In patients with poor digestive tolerance, enteral nutrition via jejunostomy should be initiated, while parenteral nutrition is reserved for those unable to tolerate enteral feeding [12,24].

As shown in the table above (Table 3), the main recommendations are derived from the World Journal of Gastrointestinal Endoscopy and the World Society of Emergency Surgery guidelines, with additional reference to national Romanian practice. While there are no dedicated European (ESGE) or American (ASGE) guidelines focused exclusively on caustic esophageal injury, both societies include relevant subsections on caustic ingestion within their broader documents on esophageal emergencies and endoscopic management. Current Romanian management largely follows these European and American standards, adapted to local clinical settings and available resources.

### 5.3. Management of Complications

Esophageal strictures

Prevention of stricture formation has been widely discussed in the literature. Prophylactic treatments such as administration of methylprednisolone (1 g/day for 3 days), mitomycin C (0.4 mg/mL) [11], or intralesional triamcinolone injections (40–100 mg/session) have been tested only in animal models, with contradictory results. Due to insufficient evidence in humans, these therapies are not recommended [7,12].

Temporary placement of esophageal stents is not routinely performed for prevention [11,25], although it may represent an alternative; however, recurrence rates reach 30–40%. Metallic, plastic, and biodegradable stents can all be used. Another preventive measure that may attenuate stricture formation is the administration of gastric antisecretory agents—for example, omeprazole 80 mg/24 h intravenously for 3 days, followed by 40 mg/24 h orally for at least 4 weeks—which has been shown to reduce caustic injury by up to 71% in Zargar IIB and IIIA lesions.

The most commonly employed method is endoscopic dilatation of strictures [12,15,18], performed with Savary-Gilliard bougies or pneumatic balloons, depending on the case. Dilatation should be initiated 3–6 weeks after ingestion, beginning with small-caliber bougies to minimize the risk of perforation. Further dilatations are then scheduled as needed, individualized in terms of frequency (generally every 1–4 weeks or less) and degree of expansion, with pneumatic balloon dilatation also being an option.

Surgical treatment is indicated when endoscopic approaches are insufficient [11]. Minimally invasive esophagectomy, using a combined thoracoscopic and laparoscopic approach, is preferred as it ensures optimal visualization and the most favorable postoperative recovery. Transhiatal esophagectomy with gastric interposition and high anastomosis is considered safe but carries a higher risk of anastomotic stenosis. The most frequently used approach remains elective partial or total esophagectomy with reconstruction and either colonic or gastric interposition, typically performed six months post-ingestion, allowing partial restoration of nutritional autonomy [24,25].

Gastric strictures

Gastric strictures are less common than esophageal strictures and typically develop later, at 4–8 weeks after ingestion, particularly in the antrum and pylorus. In rare cases, they may cause severe gastric deformity.

The first-line therapy is endoscopic balloon dilatation, which requires multiple progressive sessions. Endoscopic stenting (usually with covered metallic or biodegradable stents) can be considered in selected cases as a bridge-to-surgery option for refractory strictures, or as an alternative to surgery, though the high risk of migration significantly limits its benefit [25].

For refractory strictures without lumen, or in cases of long, multiple, or severely deforming strictures, surgical intervention remains the only option. The most frequently performed procedures are gastrojejunostomy, antrectomy with Roux-en-Y or Billroth II reconstruction, or subtotal/total gastrectomy in severe cases [7,12,24].

In all cases, nutritional support should be provided via parenteral nutrition or enteral feeding through jejunostomy, together with administration of gastric antisecretory therapy until stricture resolution [23].

Fistulas

When fistulas develop between the esophagus and adjacent structures (trachea, left main bronchus, mediastinum, pleura, pericardium, or vascular structures), the first step is discontinuation of oral intake, replacement with enteral or parenteral nutrition, and airway protection.

The first-line and curative treatment is surgical closure of the defect at both ends of the fistula, and in cases of large defects, esophagectomy with or without secondary reconstruction, combined with anti-infective and supportive therapy.

As a bridge-to-surgery approach, esophageal or dual stents may be used for temporary closure of the defect. This decision should be individualized, typically for unstable patients or those not eligible for curative surgery. In cases of vascular fistulas (e.g., aortoesophageal), an emergency endovascular approach such as TEVAR (Thoracic Endovascular Aortic Repair) may be employed. This minimally invasive technique involves placement of a stent-graft at the vascular defect site via femoral artery access under fluoroscopic guidance, and is life-saving in patients with massive hemorrhage [7,26].

Esophageal squamous cell carcinoma

Endoscopic screening is recommended beginning ten years after caustic ingestion. The interval between evaluations should be individualized, based on associated risk factors and endoscopic findings at previous examinations. Upper endoscopy may thus be performed annually, every two years, or every three years, depending on clinical context [11].

Management of patients in the previously studied cohort varied according to the severity of caustic injury. Patients with grade I lesions were managed with a liquid diet for 24–48 h and subsequently resumed normal oral intake without the need for hospitalization. Those with grade II lesions were admitted for monitoring and received parenteral nutrition alongside gastric antisecretory therapy. In patients with grade III lesions, antibiotic therapy was added to prevent secondary infections, in addition to supportive care and management of complications arising during hospitalization. Patients who developed esophageal strictures underwent weekly dilations using Savary-Gilliard bougies. Gastric strictures that could not be dilated with balloons required surgical intervention, which included repair of high or long strictures, management of acquired esophageal pseudodiverticula, treatment of fistulas, corrective esophagoplasty, repair of perforations, and surgical gastrostomy placement.

Regarding the two recently examined patients, the patient who ingested concentrated formic acid underwent serial esophageal dilations with Savary-Gilliard bougies. The patient who ingested a large volume of alkali received both serial bougie dilations and balloon dilations (CRE 6–8 mm) under radiologic guidance. This latter patient also required intensive care management for complications including pulmonary thromboembolism, pneumonia with pleural effusion, resolution of upper airway local complications, closure of the tracheostomy site, correction of anemia, and stabilization of metabolic and electrolyte imbalances.

## 6. Prognosis and Long-Term Outcomes

The prognosis correlates directly with the severity of the caustic lesions and is adversely influenced by the presence of pre-existing comorbidities at the time of ingestion [11,16].

Ingestion of substances with a pH outside the physiological tolerance range correlates with a significantly higher likelihood of severe complications, the need for operative management, prolonged hospitalization, and long-term sequelae. Mortality increases substantially when extreme pH exposure is combined with delayed presentation, extensive mucosal necrosis, or systemic toxicity.

The morbidity and mortality associated with caustic substance ingestion [2] are strongly influenced by the pH of the ingested agent, which determines the depth, extent, and progression of tissue injury. Solutions with extreme pH values (typically pH < 2 for strong acids and pH > 12 for concentrated alkalis) are responsible for the most severe clinical outcomes, due to their ability to rapidly induce necrosis and compromise the integrity of the gastrointestinal tract.

Alkaline substances (pH > 12) are particularly associated with higher morbidity because liquefactive necrosis facilitates deep tissue penetration, increasing the risk of transmural injury, perforation, mediastinitis, and extensive esophageal damage. Consequently, patients exposed to high-pH agents demonstrate increased rates of early surgical complications and are more likely to develop long-term strictures requiring repeated endoscopic or surgical interventions.

In contrast, strong acids (pH < 2) often cause intense but more surface-focused coagulative necrosis; however, gastric involvement is more pronounced, with complications such as perforation, hemorrhage, and pyloric stenosis contributing to both acute and delayed morbidity. Mortality tends to rise in cases involving large-volume ingestion or highly concentrated industrial acids.

Overall, the pH of the ingested solution functions as a key prognostic factor, guiding early risk stratification and informing clinical decisions regarding endoscopic evaluation, monitoring intensity, and early intervention

Patients presenting with Zargar grade 0–IIA lesions generally exhibit the lowest morbidity and mortality rates. In contrast, those with Zargar IIB–IIIB injuries are at high risk of developing esophageal strictures, which may lead to significant nutritional compromise and secondary protein-energy malnutrition. These patients often require repeated endoscopic dilatation sessions, which can considerably impair their overall quality of life.

In cases of Zargar grade IIIB injuries, surgical intervention is frequently necessary. These patients also exhibit a significantly increased rate of intensive care unit (ICU) admissions and a higher incidence of severe complications.

A poor prognosis is reserved for patients who develop life-threatening complications such as gastrointestinal perforation with subsequent mediastinitis or secondary peritonitis.

**Figure 2:**
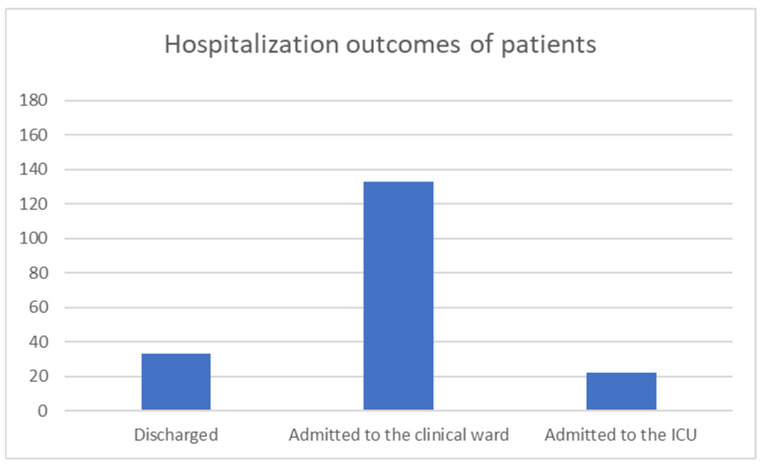
Hospitalization outcomes of patients admitted in our center.

In the previously reported retrospective study [1], the majority of patients (133 out of 188) required admission to a general ward for monitoring and specialized treatment, while 22 patients became unstable in the emergency department and were admitted directly to the intensive care unit. Of these, 18 patients died: 15 due to gastrointestinal perforation, 2 from catastrophic upper gastrointestinal bleeding, and 1 as a result of sepsis (Figure 2).

Regarding the two recently evaluated patients, both continue to experience a significantly impaired quality of life, with frequent hospitalizations, severe weight loss, and complications related to inadequate nutrition.

## 7. Case Illustrations

To meaningfully convey the complexity and multilayered evolution of post-caustic esophageal injury, it is essential not only to describe theoretical mechanisms and guideline-based management but also to contextualize these principles through real clinical experience. The following extended case illustrations integrate insights from our previous retrospective cohort of 188 patients with two recent cases encountered in our department.

Because the pathophysiology, clinical manifestations, diagnostic strategies, and therapeutic decisions discussed throughout this manuscript can only be fully appreciated when anchored in real patient trajectories, these case illustrations serve as an essential educational complement to the theoretical sections. In clinical practice, no two caustic ingestion cases evolve identically. Even when biochemical properties of the ingested agent are known, and despite the availability of endoscopic severity grading, the course of disease may shift unexpectedly, reflecting the dynamic interplay between local tissue destruction, systemic responses, comorbidities, timely medical intervention, and patient-specific vulnerabilities. This section aims to reflect that reality.

### 7.1. Cohort Foundation: Lessons from 188 Patients

Our previously published retrospective analysis of 188 patients presenting with caustic ingestion to the Clinical Emergency Hospital of Bucharest over a one-year period forms the empirical backbone of our understanding of caustic ingestion in our region. The demographic structure of this cohort revealed a striking predominance of young and middle-aged women from rural areas, with nearly 70% of ingestions being intentional. This pattern underscores the psychosocial dimensions of caustic ingestion in Romania—dimensions that significantly influence both the type of substances encountered and the timing of medical presentation.

The majority of caustic agents were alkalis, most commonly sodium hydroxide (“lye”), widely available in domestic settings. This availability aligns with our broader clinical experience: in rural households, caustic agents often remain improperly stored or transferred into unlabeled containers, increasing the risk of unintentional exposure and facilitating access for individuals in emotional distress.

From the clinical perspective, more than half of the patients underwent endoscopy within the first 24 h, allowing precise classification of mucosal injury. The distribution of lesions—ranging from superficial hyperemia to full-thickness necrosis—reflected the diversity of substances and ingestion circumstances. Of the 159 patients who underwent endoscopy, 50 exhibited grade I lesions, 82 had grade II lesions, and 27 had grade III lesions according to the Zargar classification. We can see that grade II and III injuries represented the majority of significant cases, closely correlating with the severity of complications observed during hospitalization and follow-up.

This cohort revealed several important and reproducible clinical patterns like alkali ingestion, especially involving sodium hydroxide, produced the most severe esophageal injuries with a high likelihood of transmural necrosis, perforation, and stricture formation, acid ingestion more commonly affected the stomach, leading to antral or pyloric strictures, gastric outlet obstruction, and chronic gastrointestinal dysfunction, stricture formation was the most frequent long-term complication, occurring in at least one-third of patients with moderate-to-severe lesions.

Mortality within the cohort was not negligible: 18 patients (roughly 10%) succumbed to severe complications, most commonly due to esophageal or gastric perforation leading to mediastinitis or peritonitis. These figures contextualize the seriousness of caustic ingestion beyond its immediate presentation and situate the condition as a persistent and often under-recognized public health problem.

These findings establish the clinical expectations for caustic ingestion in our region and form the benchmark against which the following two illustrative cases should be interpreted.

### 7.2. Case 1—Concentrated Formic Acid Ingestion: Silent Beginnings, Relentless Fibrosis

The first recent case, involving concentrated formic acid ingestion, illustrates the deceptive nature of acid injuries. The patient ingested a small quantity of concentrated industrial formic acid. On arrival, the patient was alert, anxious, and able to maintain airway patency, with hyperemia and erosions visible on pharyngeal inspection. Laboratory studies were normal, illustrating the well-described discordance between early clinical appearance and injury severity. Such clinical findings might falsely suggest mild injury, yet they frequently coexist with significant esophageal damage that is detectable only endoscopically.

Endoscopy within the first 24 h revealed Zargar grade IIB lesions—edema, ulcerations, and areas of mucosal sloughing extending along the esophagus. The stomach showed no significant injury, which is consistent with the pathophysiology of acid-induced coagulative necrosis: tissue proteins denature rapidly, forming a protective eschar that limits deeper penetration. As a result, acid injuries often spare the stomach relative to the esophagus, particularly when the volume ingested is small but can affect the stomach if the patients ingest large amounts of corrosive and the substance is also highly concentrated.

In the early days after ingestion, the patient maintained the ability to swallow liquids but reported increasing discomfort. This period marks the transition from acute inflammation to early healing, during which granulation tissue forms beneath the superficial necrotic layer. In acid injuries, this process often results in circumferential scarring because the mucosal injury is typically continuous rather than patchy.

Around the third week, the patient began to experience progressively severe dysphagia, now affecting even liquids. Endoscopic reassessment revealed multiple, highly rigid strictures. Dilation was initiated, but despite careful serial sessions using Savary–Gilliard bougies, luminal patency remained unstable. The esophagus demonstrated a pronounced tendency toward restenosis, consistent with the intense fibroblast activity and irregular collagen deposition seen in acid-related injuries.

Over the following months, the patient’s nutritional status deteriorated significantly. Conservative measures provided only temporary relief. Because surgical intervention was not immediately possible, long-term endoscopic management continued, with variable success. This gradual decline, punctuated by episodes of dehydration and hospitalization, closely mirrors patterns observed in our 188-patient cohort, in which late strictures and malnutrition were predominant among those with acid exposure.

### 7.3. Case 2—Large-Volume Alkali Ingestion: A Cascade of Multisystem Deterioration, Airway Injury, and Complex Complications

If the first case reveals the slow, relentless trajectory of acid-induced injury, the second case exemplifies the explosive severity of alkali ingestion. The patient had swallowed approximately 300 mL of a viscous highly concentrated alkaline household solution—a volume sufficient to overwhelm inherent mucosal defenses and initiate widespread liquefactive necrosis.

Upon arrival, the patient exhibited clear signs of upper airway involvement: stridor, drooling, progressive hoarseness, and visible mucosal sloughing. These findings necessitated immediate airway protection through orotracheal intubation, followed by tracheostomy due to extensive laryngeal edema and tissue necrosis that failed to regress adequately. This early airway compromise is characteristic of strong alkalis, whose penetration into soft tissues is rapid and profound.

Initial contrast-enhanced CT imaging demonstrated diffuse edema and wall thickening of the esophagus and stomach but no perforation. Endoscopy identified Zargar IIIB lesions, implying extensive transmural damage with areas of black necrotic tissue. From this point onward, the patient followed a turbulent clinical course punctuated by medical crises. Within days, upper gastrointestinal bleeding with hematemesis and melena emerged, moderate post-hemorrhagic anemia requiring transfusion and persistent metabolic and electrolyte disturbances. Pulmonary thromboembolism developed, complicating respiratory recovery and prolonging ICU stay. The patient also acquired nosocomial pneumonia, further challenging airway management and nutritional support. In alignment with our institutional protocol and international guidelines, enteral nutrition was deferred, intravenous proton pump inhibitors were administered, and repeat CT scans were performed during clinical deterioration.

As with acid injuries—but with greater speed and severity—the healing phase led to aggressive fibrosis. Multiple esophageal and gastric strictures formed, some so tight that dilation became technically demanding. A combination of bougie and balloon techniques was required, with radiologic guidance to minimize perforation risk. Nutritional management became increasingly difficult because oral intake was nearly impossible and enteral access was repeatedly compromised by strictures, edema, or infectious complications. The patient’s nutritional status deteriorated severely, culminating in extreme weight loss and clinical suspicion of superior mesenteric artery syndrome, prompting surgical consultation for jejunostomy feeding.

Months after the initial ingestion, the patient developed a tracheoesophageal fistula—a devastating complication that reflects deep transmural necrosis and chronic inflammation.

Despite aggressive and multidisciplinary management, the patient continued to experience poor quality of life, recurrent respiratory infections, malnutrition, and psychological distress associated with the chronicity of symptoms. This trajectory exemplifies the destructive potential of alkali agents—reinforcing the findings of our 188-patient dataset, where alkali ingestion was the primary driver of morbidity, mortality, and long-term disability.

### 7.4. Integrative Interpretation: What These Cases Tell Us About Caustic Injury

Though vastly different in tempo and anatomical distribution, the two cases present a complementary view of the natural history of caustic ingestion. The acid ingestion case underscores how deceptively mild early symptoms can evolve into severe, life-altering strictures. The alkali ingestion case demonstrates how rapidly and catastrophically tissue destruction can advance and how complications accumulate in multiplicative fashion. Also, large-volume ingestion correlates with exponentially worse outcomes regardless of agent type and nutritional failure is a central determinant of both quality of life and survival.

In both cases, the insights drawn from our original cohort—regarding demographic vulnerabilities, risk factors for severe injury, and prognosis associated with Zargar grading—were manifested vividly. Each patient’s course illustrated key concepts emphasized in the theoretical sections: the crucial importance of early presentation, the dominant prognostic role of endoscopic findings, the limitations of clinical assessment alone, the central relevance of nutritional support, and the need for coordinated, multidisciplinary management.

Ultimately, these case narratives provide more than anecdotal illustration. They offer a window into the lived clinical experience of caustic ingestion patients—an experience defined by prolonged therapeutic journeys, recurrent hospitalizations, and the persistent possibility of life-threatening complications. Their stories reaffirm the importance of prevention, early recognition, and long-term follow-up, emphasizing that caustic ingestion is not merely an acute event but a chronic, evolving disease.

These case illustrations also complement and validate the theoretical principles reviewed in earlier sections of this manuscript. They demonstrate how pH, concentration, and volume determine the depth and distribution of tissue injury; how early endoscopic grading influences but does not entirely predict clinical evolution; how nutritional compromise becomes a central determinant of morbidity; and how a multidisciplinary approach—integrating emergency medicine, gastroenterology, surgery, intensive care, ENT, radiology, and nutrition services—is indispensable for optimal management. Importantly, they show that the clinical impact of caustic ingestion extends far beyond the initial event, unfolding over months or years and requiring sustained medical, nutritional, and psychological support.

## 8. Discussion

Caustic ingestion remains a major challenge for both gastroenterologists and surgeons, due to the unpredictable evolution and the wide range of possible complications. Although the pathophysiological differences between acids and alkalis are well established—coagulative versus liquefactive necrosis—clinical outcomes depend on multiple variables, including the amount ingested, concentration, delay to presentation, and adequacy of the initial management.

Data from international literature consistently show that alkali ingestion, particularly sodium hydroxide, carries the highest risk for extensive transmural necrosis and subsequent severe strictures. Acid ingestion, while often limited to the mucosa due to coagulative necrosis, still poses a significant risk for esophageal injury and gastric outlet obstruction. Our previously published retrospective cohort of 188 patient confirmed these trends in a Romanian population, with alkali agents being the leading cause and strictures the most frequent long-term complication.

The two cases presented here emphasize the contrasting patterns of injury: one patient with concentrated formic acid developed localized but severe esophageal strictures and long-term malnutrition, whereas another patient with large-volume alkali ingestion suffered rapid deterioration, airway involvement, esophageal and gastric necrosis, repeated hospitalizations, and complex complications including esotracheal fistula. These examples illustrate the importance of early endoscopic assessment and repeated monitoring, even in patients who initially appear stable.

The importance of early endoscopic evaluation within the first 12–24 h is consistently emphasized in current guidelines, as endoscopy remains the most reliable tool for assessing mucosal injury severity. International recommendations discourage delaying the procedure unless airway compromise is present, and similar approaches were applied in our cohort. Our findings are consistent with the Zargar classification, which remains the most widely used endoscopic grading system worldwide. Comparatively, several studies have suggested that CT severity scoring may complement or even outperform endoscopy in predicting the need for surgery or the risk of perforation. In our cases, endoscopic staging guided the initial management, while CT provided essential information regarding depth of necrosis and potential complications, reflecting the multimodal diagnostic approach advocated in recent guidelines.

Management strategies remain controversial, particularly regarding the role of antibiotics, corticosteroids, and stenting for stricture prevention. Current evidence does not support systematic prophylaxis with steroids or mitomycin C, although individualized use may be considered in selected cases. Current literature remains divided regarding the use of corticosteroids, antibiotics, and acid suppression in the acute phase. Several meta-analyses have shown that corticosteroids do not reliably prevent stricture formation, whereas others suggest a potential benefit in selected grade IIb injuries. In contrast, most guidelines support the use of broad-spectrum antibiotics only in the context of suspected infection or extensive necrosis. Nutritional management is another critical component, as adequate caloric intake and prevention of catabolism influence outcomes. Enteral nutrition is generally preferred whenever feasible, with jejunostomy recommended in patients with extensive esophageal injury or prolonged dysphagia. Evidence suggests that early nutritional optimization may reduce complications and improve healing, and our clinical experience supports this perspective, particularly in complex grade III lesions. Our management strategy, tailored to the endoscopic severity and clinical evolution, mirrors this selective approach and aligns with recommendations from ESGE and WSES.

Stricture formation is reported in patients with grade IIb–III lesions. Our clinical experience aligns with these data, as both of our cases involved significant parietal injury with later luminal compromise. Endoscopic dilation remains the cornerstone for stricture management, although multiple sessions are often required. This is in line with international cohorts, where refractory strictures continue to pose therapeutic challenges.

Refractory strictures may profoundly impair oral intake. As described in several studies, this population is at substantial risk for chronic malnutrition, micronutrient deficiencies, and weight loss, often necessitating long-term enteral feeding via gastrostomy or jejunostomy. Our experience aligns with these reports, as prolonged dysphagia and nutritional compromise were key clinical concerns in our patients with high-grade injury.

Esophagotracheal and esophagomediastinal fistulas represent rare but life-threatening complications, with low incidence rates. Similarly to the cases described here, reports highlight that these complications typically occur in the context of circumferential grade III injury and deep transmural necrosis. Surgical intervention remains the definitive treatment, with outcomes heavily influenced by timing and patient stability. Our case follows this pattern, underscoring the need for early recognition and multidisciplinary management.

Additionally, patients with severe caustic damage face a markedly increased lifetime risk of esophageal squamous cell carcinoma, typically decades after the initial insult with malignant transformation often arising in areas of chronic inflammation, fibrosis, or longstanding strictures. Although the cases presented here have not yet reached this long-term interval, the evidence strongly supports the need for periodic endoscopic surveillance, especially in patients with extensive initial burns or recurrent strictures.

An essential aspect that deserves particular emphasis is the role of emergency medicine in the initial management of caustic ingestion. The first contact with the healthcare system often occurs in the emergency department, where rapid triage, airway protection, and hemodynamic stabilization are life-saving. Delays or inappropriate pre-hospital interventions, such as forced vomiting or empirical neutralization, can significantly worsen prognosis. Therefore, raising awareness among emergency physicians regarding the contraindications of nasogastric tube placement, gastric lavage, and empirical antidotes is of paramount importance. Early collaboration between emergency teams and gastroenterology, surgery, and intensive care specialists facilitates timely endoscopic evaluation and guides further management. In this regard, the emergency department represents not only the entry point but also a decisive determinant of patient outcome.

A crucial point highlighted by both our practice and international data is the need for a multidisciplinary approach. Emergency physicians, intensivists, gastroenterologists, surgeons, ENT specialists, and nutritionists must collaborate to optimize both acute and long-term outcomes. Moreover, public health interventions are urgently needed, as most voluntary ingestions occur in socioeconomically vulnerable populations with poor access to mental health support and limited awareness of the risks associated with household corrosives.

Overall, the evolution ranges from early mortality in severe presentations to chronic complications such as strictures, malnutrition, and elevated cancer risk in long-term survivors. These observations underline the importance of severity-based management strategies, structured follow-up, and multidisciplinary care throughout both the acute and chronic phases of recovery.

The limitations of our work include its narrative design and reliance on illustrative cases rather than systematic prospective data. Nevertheless, the combination of theoretical synthesis with real-world examples provides practical insights for clinicians and reinforces key messages about prevention, early recognition, and individualized care.

## 9. Conclusions and Future Directions

Caustic ingestion continues to represent a complex clinical emergency with potentially devastating short- and long-term consequences. The severity of lesions depends not only on the type and concentration of the ingested agent but also on the timeliness and appropriateness of initial emergency management. Early recognition, rapid airway protection, and hemodynamic stabilization in the emergency setting are crucial for survival and for reducing subsequent morbidity. Endoscopic assessment within the first 24 h remains the cornerstone for severity stratification and therapeutic planning. Long-term outcomes are determined by the development of strictures, fistulas, and nutritional complications, which require a multidisciplinary approach including gastroenterologists, surgeons, emergency physicians, ENT specialists, and nutrition experts.

From a public health perspective, prevention through education, safe storage of corrosives, and psychosocial support remains essential. Overall, integrating emergency medicine principles with multidisciplinary long-term care offers the best chance to improve prognosis and quality of life in patients with post-caustic esophagitis.

Overall, integrating data from previous and recent cases demonstrates that early recognition, stratified management, and vigilant follow-up are critical to reducing complications and improving both short- and long-term prognosis in patients with caustic ingestion.

Beyond the current therapeutic strategies, several future directions should be considered in order to improve outcomes in patients with post-caustic esophagitis. Research efforts need to focus on early biomarkers capable of predicting complications, innovative antifibrotic and regenerative therapies aimed at reducing stricture formation and the development of minimally invasive endoscopic technologies with greater efficacy and safety. Furthermore, international consensus guidelines and structure long-term surveillance programs are essential to harmonize management approaches and ensure early detection of esophageal carcinoma. Finally, stronger public health interventions, including education, regulatory measures and targeted psychosocial support, remain crucial in reducing both the incidence and the devastating impact of caustic ingestions.

## Figures and Tables

**Table 1:** The most commonly ingested corrosive agents [8].

The Most Commonly Ingested Corrosive Agents	
Acids	Alkali
Hydrochloric acid	Sodium hydroxide
Chromic acid	Potassium hydroxide
Phosphoric acid	Calcium hydroxide
Nitric acid	Ammonium hydroxide
Sulfuric acid	Lithium hydroxide
Formic acid	Sodium hypochlorite
Acetic acid	Sodium tripolyphosphate
Hydrofluoric acid	

**Table 2:** The Zargar Classification and its predictive value.

The Zargar Classification and Its Predictive Value			
Grade	Endoscopic Findings	Risk of Complications	Mortality
0	No lesions	None	Low
I	Edema and hyperemia of the mucosa	None	Low
IIA	Superficial localized erosions, blisters and/or ulcers with or without hemorrhage, friability of the mucosa	None or minimal local complications like low debit or autolimited hemorrhage	Low
IIB	Deeper lesions with focal or circumferential distribution	Gastrointestinal hemorrhage Strictures Fistulas Esophageal carcinoma Systemic complications associated with acidosis, infections, malnutrition and shock	Medium
IIIA	Multiple deep ulcers with grey, brown or black discolorations and focal necrosis		Medium–High
IIIB	Extensive necrosis		High
IV	Perforation	All of the above and even death	Highest

**Table 3:** Comparison of international guidelines on the management of caustic esophageal injury—recommendations summarized from WJGE and WSES guidelines, complemented by Romanian practice.

Comparison of International Guidelines on the Management of Caustic Esophageal Injury						
Guideline/Society	Timing of Endoscopy	CT Role	Use of Corticosteroids	Antibiotics	Stricture Prevention	Nutrition Support
WJGE (World Journal of Gastrointestinal Endoscopy)	Within 24 h (≤48 h max)	Recommended in suspected transmural necrosis. Useful for surgical decision	Not routinely recommended. In case of use, intralesional steroid injection prior to bougie dilatation could improve dilatation and reduce the number of sessions needed	Only if infection	No strong evidence for prophylaxis. Esophageal SEMS is considered a last resource with best recommandations for biodegradable stents	Early enteral feeding preferred
WSES (World Society of Emergency Surgery, 2019)	12–24 h in stable patients	Mandatory for perforation suspicion	Not recommended routinely	Empirical in severe cases	Stents not recommended for prevention	Start oral feeding when safe
Romanian National practicies	Endoscopy in <24 h	CT for complications	Avoid steroids	Antibiotics in selected cases	Endoscopic dilation mainstay	Individualized based on tolerance

## Data Availability

No new data were created or analyzed in this study.
